# Electrical Performance Analysis of High-Speed Interconnection and Power Delivery Network (PDN) in Low-Loss Glass Substrate-Based Interposers

**DOI:** 10.3390/mi14101880

**Published:** 2023-09-29

**Authors:** Youngwoo Kim

**Affiliations:** Department of Semiconductor System Engineering, Sejong University, Seoul 05006, Republic of Korea; youngwoo@sejong.ac.kr; Tel.: +82-2-3506-3408

**Keywords:** glass substrate, electrical performance, high-speed interconnection, power delivery network, signal/power integrity

## Abstract

In this article, electrical performance analysis of high-speed interconnection and power delivery network (PDN) in low-loss glass substrate-based interposers is conducted considering signal integrity (SI) and power integrity (PI). The low-loss glass substrate is a superior alternative to silicon substrate in terms of high-speed signaling and fabrication yield. However, the low-loss of the substrate is vulnerable to power/ground noise in the PDN since the low-loss property of the substrate cannot suppress the noise naturally. In this article, an in-depth electrical performance analysis is conducted based on various measurements and simulations to fully benefit the advantages of the low-loss glass substrate. First, the fabrication process and test vehicles for the analysis are explained. Using the test vehicles, the electrical performance of the glass interposer’s high-speed interconnection is compared with those of silicon and organic interposers. The insertion loss, eye-diagrams, and signal bandwidths of three interposer channels are compared and analyzed based on electromagnetic (EM) and circuit simulations. Also, the electrical performance of the through glass via (TGV) channel is measured and compared with through silicon via (TSV) channel. The high-speed interconnection of the glass interposer showed better performance for most of the parameters which is more suitable for maintaining the SI. Even though the low-loss of the glass substrate ensured the SI, power/ground noise issues in the PDN must be analyzed and solved. In this article, various cases inducing the power/ground noise in the PDN are considered, simulated, and measured. To solve the issues, ground TGV design and electromagnetic bandgap (EBG) design are proposed for an efficient broadband suppression of the noise generated in the glass interposer PDN.

## 1. Introduction

**Figure 1:**
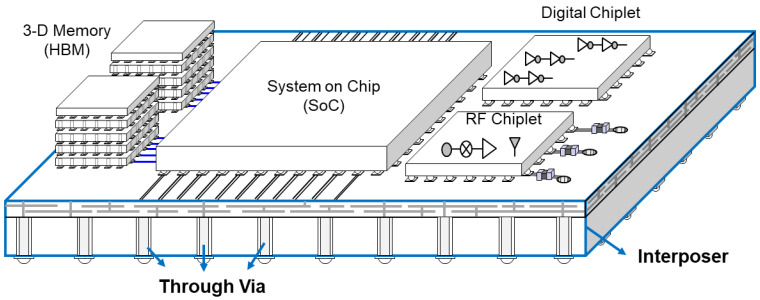
Conceptual image of 2.5-D integrated system with interposer is depicted. Electrical performance is significantly affected by the material properties and design rules of the interposer.

Recently, the realization of high-speed electrical systems with wide bandwidths, low power consumption, small form factors, and low manufacturing costs has been a continuous challenge. In addition, 2.5-dimensional (-D) integration based on through silicon via (TSV) and silicon interposers has gained substantial attention as a promising solution toward industrial challenges due to their compact design and fabrication process readiness [1,2,3,4,5,6,7,8]. The TSV technology enables vertical interconnection between homogeneous or heterogeneous integrated circuits (ICs) which provide much shorter interconnection length compared to the conventional lateral integration. The silicon interposer technology increases ICs’ integration density and the number of channels since it allows very fine pitch metallization [9]. Because of these merits, 2.5-D integration based on both TSV and silicon interposer technology increases the system bandwidth significantly [10]. Figure 1 depicts a conceptual image of a 2.5-D integrated system with an interposer. Even though the silicon interposer-based 2.5-D integration provides various advantages compared to the 2-D integration, the manufacturing cost still remains high due to the following reasons: limited wafer dimension (12-inch) and additional fabrication steps required to isolate the conductors from the conductive silicon substrate. The conductivity of the silicon substrate can cause significant signal integrity (SI) issues at higher operating frequencies [11]. 

To mitigate these problems, a glass substrate is proposed for interposers. The glass substrate has several advantages, namely excellent dimensional stability, closely-matched coefficient of thermal expansion (CTE) to silicon dies to be assembled, availability of glass substrates in large and thin panel sizes compared to that of silicon wafers, and excellent electrical resistivity of the glass substrate that allows to low signal loss up to GHz range [12]. Therefore, 2.5-D integration based on glass interposer is a potential means of achieving high-bandwidth and high-integration density electrical systems with reduced manufacturing cost. 

**Figure 2:**
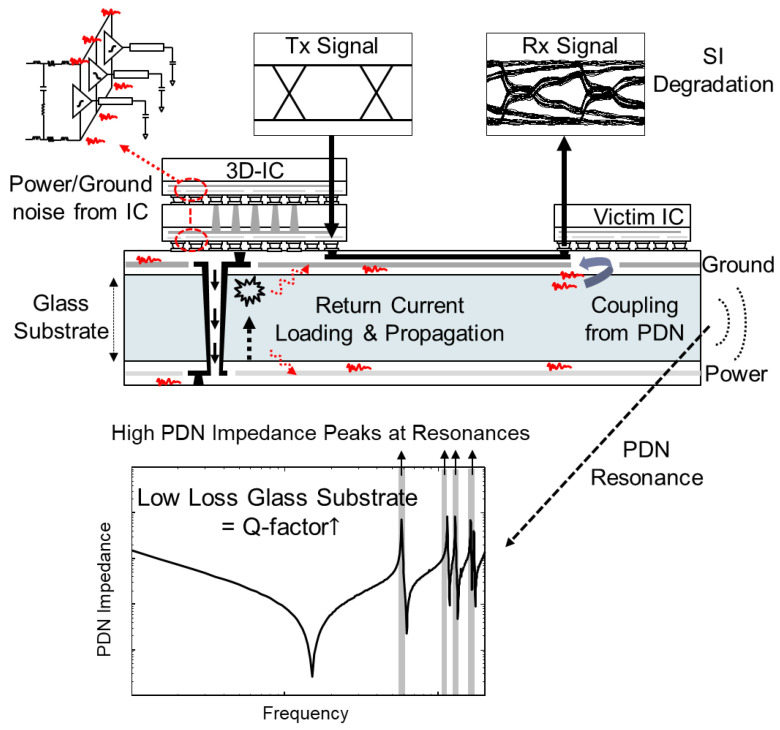
Power/ground noise issues in the glass interposer PDN are depicted. When the noise (red lines) is induced in the PDN, low-loss of the glass substrate cannot suppress the noise propagation. These issues must be solved to fully benefit from the advantages of the low-loss glass substrate.

In spite of these advantages, the low-loss of the glass substrate is vulnerable to power/ground noise in the power delivery network (PDN) since the low-loss property of the substrate cannot suppress the induced noise naturally [13,14,15,16]. When the power/ground noise is induced in the PDN due to simultaneous switching noise (SSN), PDN resonance generates a large PDN impedance at certain frequencies, and the return current is affected. As a result, various signal integrity (SI) and power integrity (PI) issues can arise. In particular, when the noise couples to vertical interconnects such as through glass vias (TGVs), the SI/PI can be degraded even more at solder joints and micro-bumps [17,18] underneath TGVs due to dimensional mismatches. Various types of noise sources that can cause power/ground noise in the glass interposer PDN are depicted in Figure 2. 

Power/ground noise is becoming more serious in heterogeneous 2.5-D systems with various mixed applications. Therefore, the power/ground issues in the PDN of the glass interposer must be thoroughly analyzed and solved to maximize the advantages of the glass substrate. Also, in order to accommodate various digital, analog, and beyond 5G RF applications, the SI/PI must be maintained in broadband frequency ranges. Therefore, power/ground noise suppression in the glass interposer should also be conducted in the broadband frequency ranges.

In this article, an in-depth electrical performance analysis is conducted based on various test vehicle measurements and simulations to fully benefit from the advantages of the low-loss glass substrate. First, the fabrication process and test vehicles for the analysis are explained. Based on the fabrication process, design rules for test vehicle design/fabrication and simulation pattern designs are determined. In the following section, using the test vehicles, the electrical performance of the glass interposer’s high-speed interconnection is compared with those of silicon and organic interposers. The insertion loss, eye-diagram, and signal bandwidth of three interposer channels are compared and analyzed. Based on the frequency domain simulation results, eye-diagram simulations and analysis in the time domain are conducted. The high-speed interconnection in the glass interposer showed a larger eye-opening voltage and smaller timing jitter than other interposers. Capacitance and conductance of the glass and silicon channels used to determine factors affecting the loss profile are thoroughly analyzed. The conductivity of the silicon substrate contributed to the high insertion loss of the high-speed interconnection in the silicon interposer. This high conductivity also affected via-to-via noise coupling. The via-to-via noise coupling between the TSV and through glass via (TGV) were compared and measured using the fabricated test vehicles. Overall, the high-speed interconnection in the glass interposer showed superior electrical performance than silicon and organic interposers.

To define and prove the power/ground noise issues, various cases inducing the power/ground noise in the PDN were considered, simulated, and measured. Impedance profiles of the interposer PDNs are compared. The noise propagation and coupling issues are also compared and analyzed. The glass interposer PDN is severely deteriorated by the noise in the PDN. To solve the issues, ground TGV design and electromagnetic bandgap (EBG) design are proposed for an efficient and broadband suppression of the noise generated in the glass interposer PDN. Glass interposers have many advantages, and they are suitable for advanced semiconductor packaging. In order to fully utilize these advantages, solving noise issues in the glass interposer PDN is mandatory.

## 2. Fabrication Processes and Design Rules of the Glass Interposer Test Vehicles for Measurements and Simulations

This section explains the fabrication processes and design rules of the glass interposer test vehicles to be fabricated, simulated, and measured. In this study, double-sided glass interposer test vehicles were designed including the substrate core and two metal layers on the top and bottom of the substrate. As a result, there were a total of four metal layers. The glass substrate was 100 μm thick and the polymer layers were between the glass substrate and metal layers. For the polymer layers, organic dry films were used as build-up dielectrics because of their low dielectric loss, smooth surface, and ease of panel processing. Also, the average roughness of the film after the desmear process was controlled to enable high precision fine-line patterning and to reduce the signal loss at high frequencies due to the skin effects. Another notable attribute of this film is the low water absorption, which is essential for stable high-frequency performance. The silicon-like low CTE and high modulus attributes of glass prevented significant warpage and enabled substrate flatness after each assembly process. Freshly drawn glass itself is a very strong material but the through glass via (TGV) formation and dicing processes generate defects in the glass which dramatically weaken the strength of the glass. The low-loss polymer was laminated on both sides of the glass substrate to build up the metal layers and, at the same time, to prevent glass cracking [19,20].

**Figure 3:**
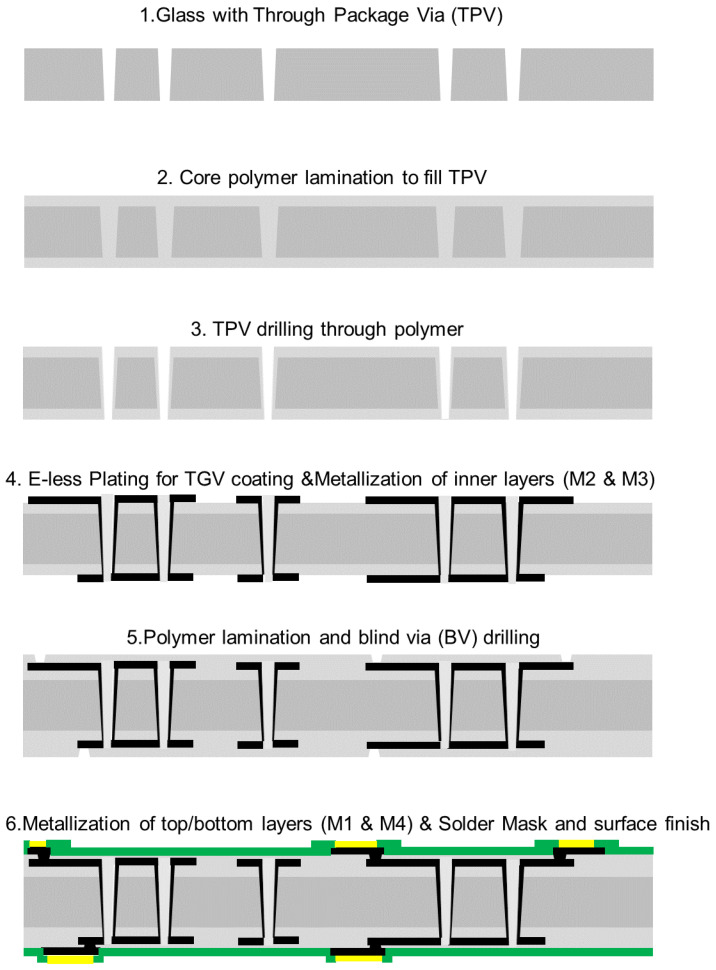
Fabrication processes are described step-by-step.

A low-cost process flow for panel-level glass substrate fabrication was developed to realize glass interposers with four metal layers, as illustrated in Figure 3. The glass substrate with 100 μm thickness was processed at 6 inch × 6-inch panel size. The fabrication processes are scalable to 500 mm × 500 mm for potential low-cost and high-volume manufacturing. The glass substrate fabrication started with the formation of 100 μm through vias in the glass core. After cleaning the glass substrate, the glass surfaces were then coated with silane coupling agents to prepare them for polymer lamination. Dry-film polymers were simultaneously laminated onto both sides of the glass substrate. Vacuum hot-press was performed at 100 °C for 60–90 s. This is followed by curing at 130 °C for 60 min. During the curing process, the polymers attained a low viscosity that allowed them to flow and completely fill the through vias. Laser ablation was then applied to drill through the polymer-filled glass vias and re-open them at a slightly smaller diameter to accommodate the alignment tolerance of the laser-drilling system. The polymer layer offered several benefits including enhanced handling of ultra-thin glass substrates for large-scale manufacturing, stress buffering between plated copper and glass for improved reliability and reducing the signal loss due to the low-loss tangent of the polymer.

The polymer coating on the glass surface aided in high-throughput electro-less (E-less) copper seed layer deposition onto the substrates with good adhesion. Ultraviolet (UV) lithography and semi-additive processing (SAP) were used to metalize the inner circuitry layers M2 and M3 with precision. The same lamination and metallization processes were followed for both the inner M2 and M3 and outer M1 and M4 circuitry layer patterning. The vias between the inner and outer metal layers (micro-via) were formed by a UV laser ablation process. 

**Figure 4:**
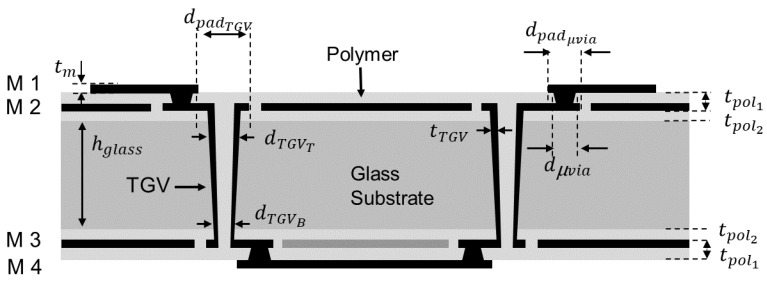
Cross-sectional view of the daisy chain structure is shown to explain important structures.

Figure 4 shows the cross-sectional view of the glass interposer test vehicle that is designed and fabricated for this study to explain the structures and design rules in more detail. It is a daisy chain structure with TGVs, micro-vias, transmission lines referencing power/ground planes. The physical dimensions and material properties of the test vehicles are summarized in Table 1. The height of the glass substrate (*h_glass_*) is 100 μm and the thickness of the polymer layer 1 and 2 ($t_{pol_{1}}$, $t_{pol_{2}}$) were 35 μm and 17.5 μm, respectively. The thickness of the metal layer (*t_m_*) was 10 μm, except for M4 layer, which turned out to have a thickness of approximately 7 μm due to process variation during electro-less (E-less) plating. Due to the unique through substrate via fabrication processes, TGVs are tapered with different top and bottom diameters. The top and bottom diameters of the TGV (*d_TGVT_* and *d_TGVB_*) and TGV pad (*d_padTGV_*) were 100 μm, 60 μm and 120 μm. respectively.

**Figure 5:**
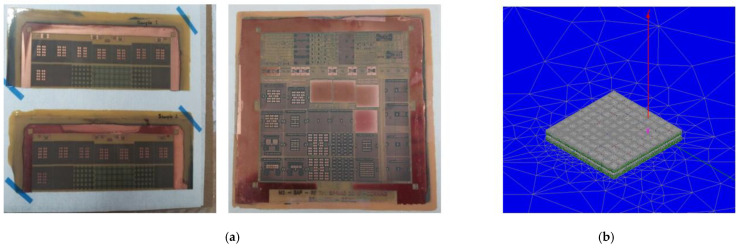
(**a**) Fabricated coupons including various test vehicles to be measured and analyzed are shown. (**b**) The designed test vehicle is being analyzed using the 3-D EM simulator, HFSS.

In the case of the TGV, only the sidewall was filled with copper and the thickness (*t_TGV_*) was approximately 12 μm to 15 μm. The diameter of the micro-via ($d_{\mu via}$) and micro-via pad ($d_{pad_{\mu via}}$) were 45 μm and 75 μm, respectively. The relative permittivity of the glass substrate (ε*_glass_*) and polymer (ε*_pol_*) were 5.3 (at 2.4 GHz) and 3 (at 10 GHz), respectively with a loss tangent (*tan* δ*_glass_*, *tan* δ*_pol_*) of 0.004 (at 2.4 GHz) and 0.005 (at 10 GHz). The designed glass interposer structure for the study and analysis shown in the following sections has the same physical dimensions and material properties shown in this section. A more detailed explanation of the designed structures will be given in following sections. Fabricated coupons, including test vehicles to be measured and analyzed, are shown in Figure 5a. These structures are also analyzed using the 3-D electromagnetic (EM) simulator so that they can be compared with silicon and organic interposers. In Figure 5b, the designed structure is imported to the 3-D EM simulator, HFSS. Detailed simulation set-up, boundaries, and port conditions are summarized in Appendix A. 

## 3. Electrical Performance Analysis and Comparison of High-Speed Interconnections

### 3.1. Interposer Channel Analysis and Comparison Based on Simulation

**Figure 6:**
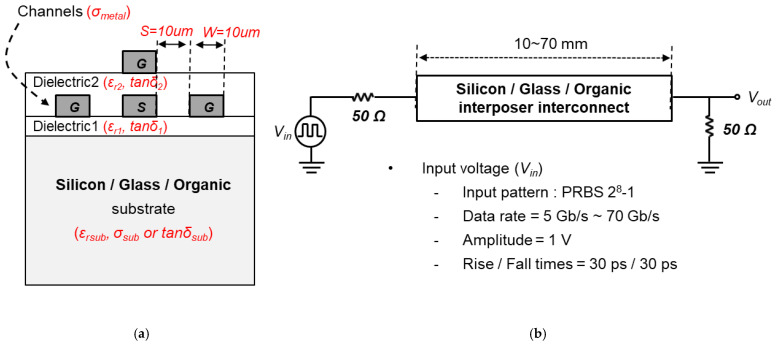
(**a**) Cross-sectional view of silicon, glass and organic interposer channel and (**b**) Simplified model of the interposer channel for time domain simulation.

In this section, the electrical performance of the interposer channels is analyzed and compared based on EM and circuit simulations. The glass interposer channel is compared with silicon and organic interposer channels. First, the insertion loss of the interposer channels with different substrate and build-up layer materials are simulated. Figure 6a shows the cross-sectional view of the interposer channels to be analyzed and Figure 6b shows a simplified model of the interposer channels for the time domain simulation. The transmitter (Tx) and receiver (Rx) are modeled by the series resistance of 50 Ω. The input signal ($V_{in}$) is a pseudo-random bit sequence of $2^{8}-1$, with a rise- and fall-time of 30 ps. The baseline data rate and the amplitude of the input signal are 10 Gb/s and 1 V, respectively.

Table 2 is a summary of material properties for the silicon and organic interposers. Channels, build-up layers, and substrate heights are set to be identical to the values shown in Table 1 for all interposers. Line width and spacing of the interposer channels are set to be 10 μm. The CPW line structure has been chosen for analysis and comparison since the electrical field and magnetic field of the CPW line penetrate into the interposer substrate. For this reason, channel performance is directly affected by the interposer substrate.

**Figure 7:**
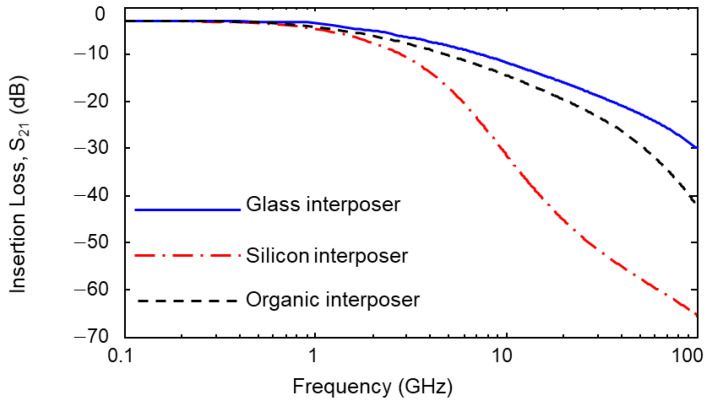
Simulated insertion losses ($S_{21}$) of the interposer channels are plotted (length = 70 mm).

**Figure 8:**
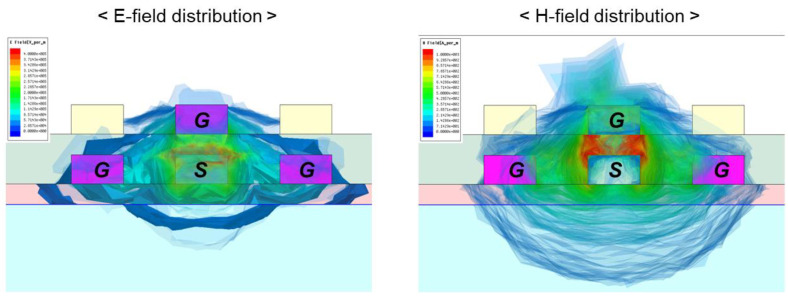
Simulated electric and magnetic fields in the interposer channel are shown. Since they penetrate into the interposer substrate, channel properties are affected by the material properties of the interposer substrate and build-up layer.

The simulated insertion losses ($S_{21}$) of the glass, silicon, and organic interposer channel are plotted in Figure 7. 3-D EM simulations are conducted up to 100 GHz. Since copper is used to realize channels for three interposers, the conductor losses of each interposer are the same, resulting in the same $S_{21}$ level at the low frequency range. At the frequency range over GHz, the $S_{21}$ levels of the interposer channels begin to fall from the different frequencies depending on the dielectric losses of the substrates and build-up layers. The overall insertion loss of the silicon interposer channel is much larger than those of glass and organic interposer channels due to the lossy silicon substrate. The overall insertion loss level of the glass interposer channel is lower than that of the organic interposer because the loss tangents of the organic interposer’s substrate and build-up layer are higher than those of the glass interposer as shown in Table 1 and Table 2. Figure 8 shows electric and magnetic fields in the interposer channel. As can be seen in Figure 8, both electric and magnetic fields penetrate into the substrate. Channel properties are affected by the material properties of the interposer substrate and build-up layer. 

In the case of the glass and organic interposers, the fluctuation of the reflection is observed in the insertion loss curves whereas the insertion loss of the silicon interposer has no reflection impact. It is due to the attenuation levels of the glass and organic interposer channels being low that the amount of the reflected waves that are propagated are considerable in the channel. In contrast, the reflected waves in the silicon interposer channel are significantly attenuated by the silicon substrate, resulting in the insertion loss curve without the fluctuation generated by the reflections. 

**Figure 9:**
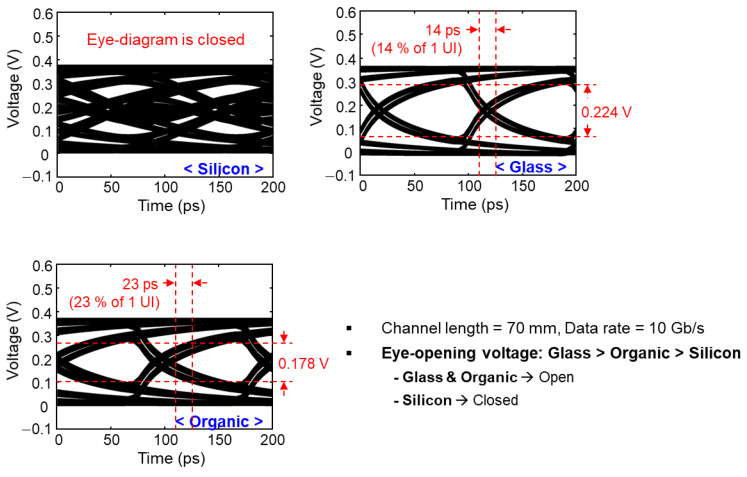
Simulated eye-diagrams are shown with eye-opening voltages and timing jitters. In this case, long interposer channels (70 mm) with the data rate of 10 Gb/s were targeted.

**Figure 10:**
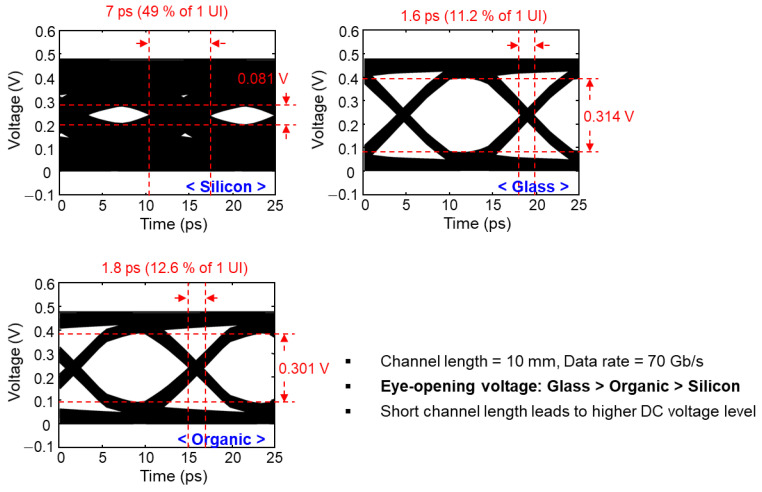
Simulated eye-diagrams are shown with eye-opening voltages and timing jitters. In this case, shorter interposer channels (10 mm) with the data rate of 70 Gb/s were targeted.

Eye-diagram simulations were conducted in the time domain based on the S-parameters of the channels that are obtained using 3-D EM simulations. In the insertion loss simulation (Figure 7), 70 mm interposer channels were simulated. In order to conduct various eye-diagram simulations, additional interposer channels were simulated starting from 5 mm and increasing to 70 mm. The eye-diagram simulation set-ups such as input voltage amplitude, rise/fall times, data rate and patterns are summarized in Figure 6b. In Figure 9 and Figure 10, eye-diagrams of three channels are compared as representatives. Opening voltages and timing jitters are also marked in the figures for easier comparisons. In Figure 9, relatively long interposer channels (70 mm) are analyzed with an input data rate of 10 Gb/s. In this case, the eye-diagram is closed for the silicon interposer channel and glass interposer channel showed the largest eye-opening voltage and the smallest timing jitter. 

In Figure 10, relatively shorter interposer channels (10 mm) are targeted with an input of higher data rate, 70 Gb/s and being analyzed. In this case, the silicon interposer channel has very small eye-opening voltage with large timing jitter. Still, the glass interposer channel showed the largest eye-opening voltage and the smallest timing jitter. 

**Figure 11:**
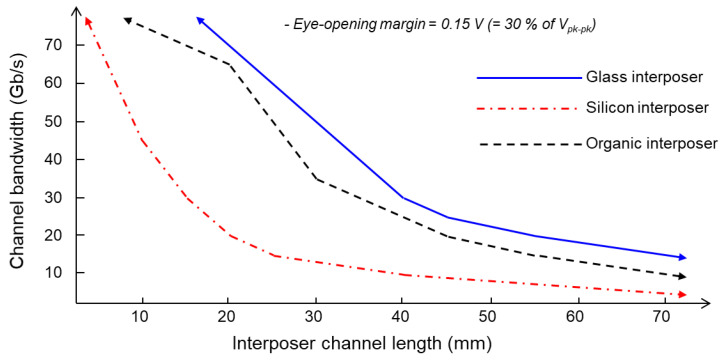
Channel bandwidth of interposer channels are derived based on eye-diagram simulations.

Various eye-diagram simulations with different interposer channel lengths and data rates were conducted. Figure 11 shows the channel bandwidth of the interposer channels with different channel lengths and data rates obtained from the eye-diagram simulation results. At a given interposer channel length, a data rate which can support signal transfer with eye-opening margin is determined (targeted to be 30% of the peak-to-peak voltage in this study). As can be seen from Figure 11, the glass interposer can transmit signal with higher data rate than silicon and organic interposers. 

### 3.2. Through Via Characterization and Comparison between Through Glass Via (TGV) and Through Silicon Via (TSV) Based on Measurements

**Figure 12:**
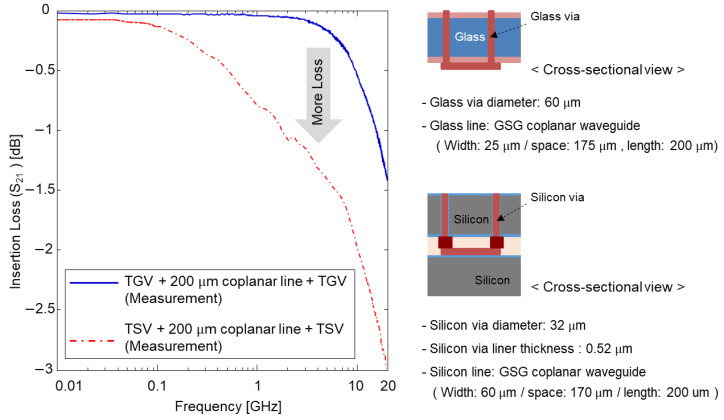
Measured insertion losses ($S_{21}$) of the TGV and TSV channels are compared. The dimensions of the test vehicles for measurements are also summarized.

**Figure 13:**
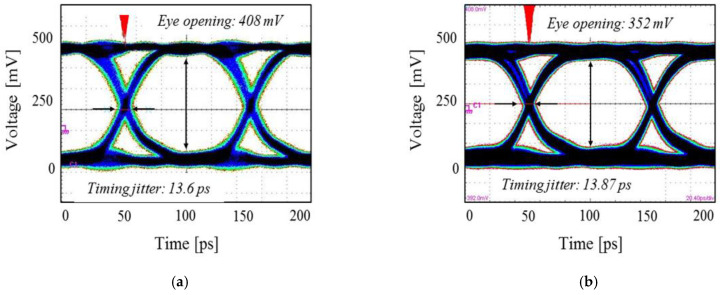
Measured eye-diagrams of the TGV and TSV channels are compared. Eye-diagrams of the (**a**) TGV channel and (**b**) TSV channel are shown with eye-opening voltages and timing jitters.

As summarized in the previous sub-section, the glass interposer channel showed low loss characteristics up to the high-frequency range, showed the largest eye-opening voltage, and showed the smallest timing jitter. On the other hand, the silicon interposer channel showed the worst channel characteristics. In this section, the electrical performance of the TGV and TSV is characterized and compared based on the measurement. The TSV test vehicles were designed and fabricated for the measurement. In order to measure test vehicles using GSG type micro-probes, and test vehicles using two GSG through via pairs and GSG CPW line which interconnects two through via pairs were designed and fabricated. As a result, the test vehicles were measured in the same plane. The test vehicles included the GSG interposer channel, but the electrical performance was dominated by the via properties. Figure 12 compares the measured insertion losses of the TGV and TSV channels. Figure 13 compares measured eye-diagrams of the TGV and TSV channels [21,22,23] ($2^{31}-1$ PRBS signal with a data rate of 10 Gb/s & 90 cm high-frequency cable has −1.3 dB insertion loss at 10 GHz).

The low loss of the glass substrate contributed to lower channel loss profile over the GHz range. In addition, the eye-opening voltage of the TGV channel is larger than that of TSV channel and the timing jitter of the TGV channel was smaller than that of TSV channel. 

**Figure 14:**
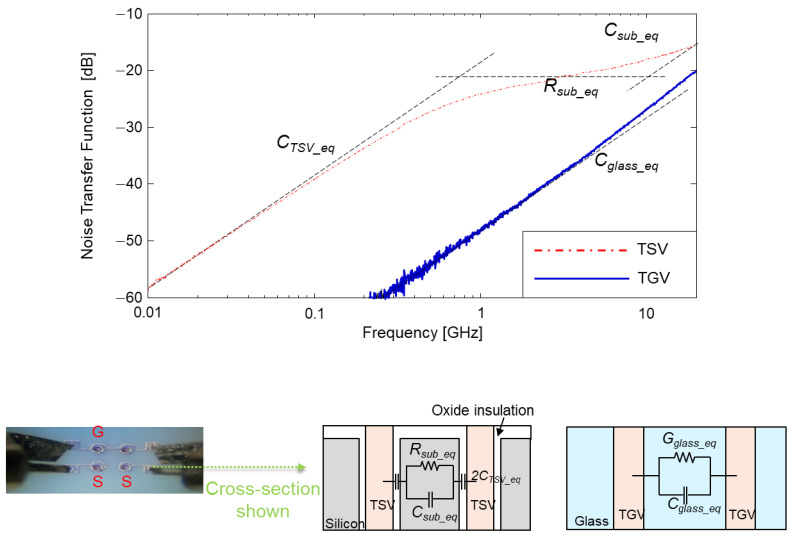
Noise couplings between TSV-to-TSV and TGV-to-TGV are measured and compared. Parameters that dominate noise-coupling profiles are also marked in the graph.

**Figure 15:**
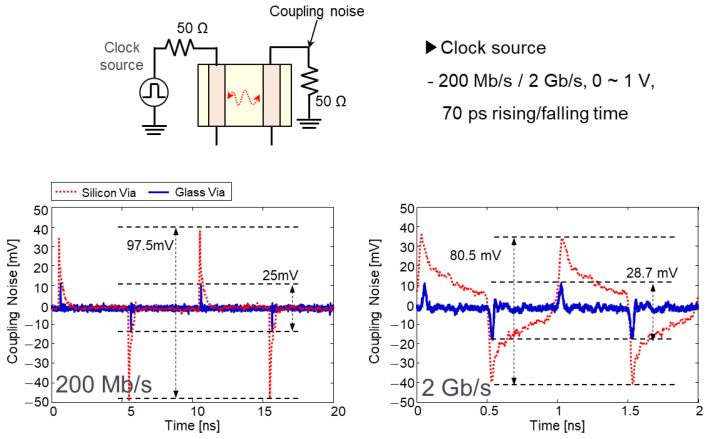
Noise couplings between TSV-to-TSV and TGV-to-TGV are measured and compared in the time domain. Different clock signals are injected (200 Mb/s and 2 Gb/s) for the comparison.

Through via to trough via noise couplings in the glass and silicon interposers were also measured and compared in the frequency domain (Figure 14) and in the time domain (Figure 15). For the measurement, GS patterns were fabricated [15,21,22,23]. The top-side probing image is included in Figure 14. In Figure 14, parameters that dominate the noise-coupling profiles are also marked. Since the silicon substrate has higher relative permittivity than that of the glass substrate, it increases capacitance between TSVs, providing lower impedance coupling path. For this reason, noise coupling between TSV-to-TSV is more severe than noise coupling between TGV-to-TGV.

Clock sources were injected into the test vehicles to measure coupled noise in the time domain. Input conditions for the clock source are described in Figure 15. The noise is coupled to adjacent through via at the rising and falling edge of the clock input signal, which has very high-frequency components. The coupled voltage noise level is much smaller in the case of the TGV-to-TGV noise coupling than that of TSV-to-TSV noise coupling. 

In this section, channel properties of the glass interposer were measured, simulated, and compared with those of silicon and organic interposers. Glass interposer channel maintained the best signaling performance compared to silicon and organic interposer channels. The glass interposer channel supports signaling to a several GHz range, which is suitable for high bandwidth applications, mixed-signals integration, mm-wave applications, and even 5 G applications. 

In the following section, power/ground noise issues in the PDN are reported with possible solutions to maximize the advantages of the glass substrate. 

## 4. Power/Ground Noise Issues in the Glass Interposer PDN and Solutions

### 4.1. PDN Impedance Analysis and Comparison

**Figure 16:**
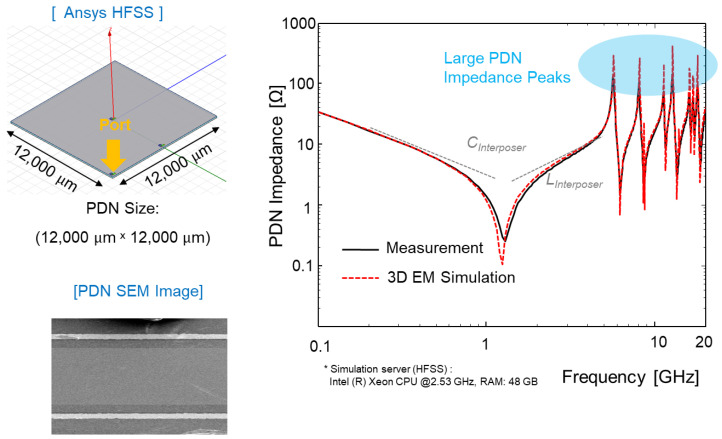
Measured glass interposer PDN impedance is compared with simulation result.

Glass interposer-based 2.5-D system integration is a potential means of realizing cost-effective and high-performance electrical systems. However, in spite of these advantages, glass interposers suffer from PDN resonance issues stemming from the low loss of the glass substrate [24,25]. Measured glass interposer PDN impedance is compared with 3-D EM simulation result in Figure 16.

As can be seen in Figure 16, the measured PDN impedance showed good correlation with that of the simulation result. At the PDN mode resonance frequencies, large impedance peaks were generated. The low-loss substrate was equivalent to the substrate with a high Q-factor. As a result, sharp impedance peaks were generated in the glass interposer PDN. 

**Figure 17:**
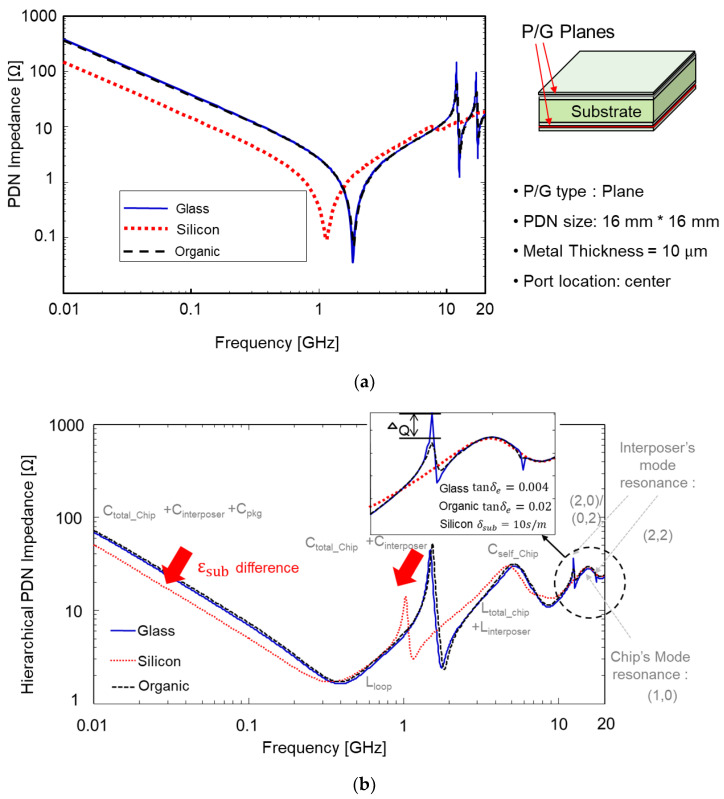
(**a**) Simulated PDN impedances are compared. (**b**) Hierarchical PDN impedances are compared considering chip-interposer-package structure. In this case, the sharp peaks are still visible.

In Figure 17, PDN impedances are compared using the 3-D EM simulation for the glass, silicon, and organic interposer PDNs. First, doubled-sided interposer PDNs are targeted and compared in Figure 17a. Compared to other PDNs, the glass interposer PDN showed the sharpest impedance peaks in the frequency domain. In Figure 17b, hierarchical PDN impedances are compared assuming a chip-interposer-package structure. Even with the on-chip and package PDN, sharp impedance peaks were still observed for the glass interposer PDN. These peaks may cause PI and SI issues. In particular, large PDN impedance affects return a current of the signal TGV penetrating the PDN. These impacts will be deeply discussed in the following sub-section. 

### 4.2. Return Current Loading and PDN Induced Crosstalk Issues

When the power/ground noise is generated in the glass interposer PDN the low loss of the glass substrate cannot suppress the generated noise, and it becomes a potential source of the SI/PI degradation. Not only the activities of circuits sharing the PDN, but also the return current of the signal vias penetrating the PDN can induce noise in the PDN. As can be seen from Figure 16 and Figure 17, the PDN impedance of the glass interposer remain mostly low up to 20 GHz except mode resonance frequencies. It indicates that the PDN serve as a good return current path for the signal TGV except mode resonance frequencies. At mode resonance frequencies, the PDN impedance increases dramatically, and the return current can be loaded to the PDN. As a result, the insertion loss of the signal TGV can increase at these frequencies leading to SI problems. Also, the loaded noise in the PDN propagates as a power/ground noise wave causing PI issues or it becomes a source of the PDN-induced crosstalk when it couples to other signal vias or high-speed interconnects adjacent to the PDN [26]. In this subsection, the return current loading issues in the glass interposer PDN associated with mode resonances are verified based on measurements. 

**Figure 18:**
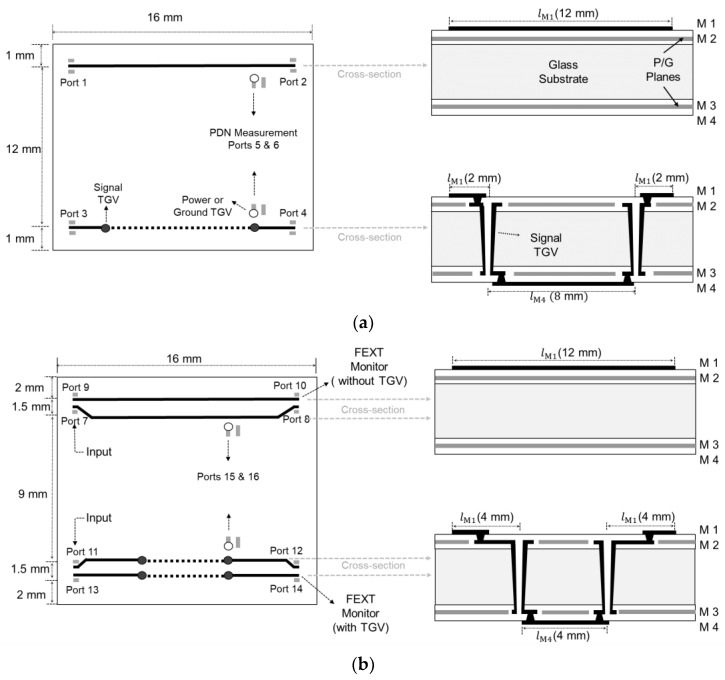
Fabricated test vehicles are shown. (**a**) Test vehicle for analyzing the insertion loss and the PDN impedance is shown. (**b**) Test vehicle for analyzing the PDN induced crosstalk is shown.

The designed and fabricated test vehicles are depicted in Figure 18. A total of two test vehicles were fabricated and measured in both the time and frequency domains. Figure 18a shows the top and cross-sectional view of the test vehicle for analyzing the insertion loss and the PDN impedance. Figure 18b is fabricated to analyze the PDN induced crosstalk. In Figure 18, the port (measurement pad) numbers and locations are marked.

**Figure 19:**
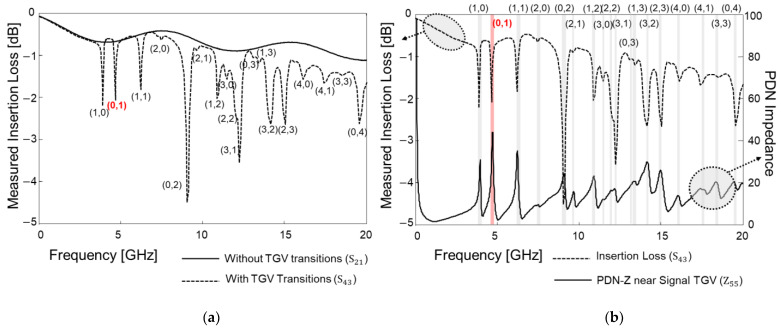
The test vehicle shown in Figure 18a is measured. (**a**) Insertion losses are compared without and with TGV transitions penetrating the PDN. (**b**) The insertion loss with TGV transitions is compared with the PDN impedance measured near the TGV transition (port 5). The (0,1) mode frequency is analyzed in Figure 19, and it is marked with red for the visibility.

In Figure 19, the measurement results for the test vehicle explained in Figure 18a are plotted. The insertion losses of the glass interposer channel with and without the TGV transitions penetrating the PDN are compared in Figure 19a. The measurement has been conducted up to 20 GHz and the insertion loss profile remains similar for both cases except for PDN mode resonance frequencies. This phenomenon becomes more apparent by comparing the insertion loss and the PDN impedance which is shown in Figure 19b. The PDN impedance near the signal TGV has a low impedance profile of up to 20 GHz; therefore, the PDN serves as a good return path excluding the PDN mode resonance frequencies. In the glass interposer, the return current of the signal TGV is severely affected by the mode resonances than other PDN factors. At the mode resonance frequencies where high PDN impedance peaks are generated, the return current of the channel is affected directly since the high impedance PDN takes more power than the receiver. The low loss of the glass substrate (equivalent to high Q-factor) generates sharp PDN impedance peaks affecting the return current of the TGV channel. The magnitude of these impedance peaks is determined by the loss tangent of the glass substrate. For this reason, the return current is discontinued, and the insertion loss increases significantly for the channel with TGV transitions. At these frequencies, signal quality at the receiver side is expected to be degraded. The appearance of high impedance peaks at these frequencies depends on the physical dimensions of the PDN, locations of the signal TGV, and material properties of the glass substrate and polymer.

**Figure 20:**
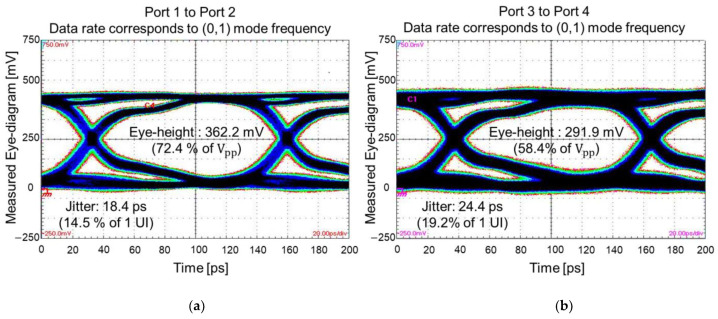
Eye-diagrams are measured and compared. As an input, the data rate that corresponds to (0,1) mode of the PDN is selected. (**a**) Eye-diagram of the glass interposer channel without TGV transitions is plotted. (**b**) Eye-diagram of the glass interposer channel with TGV transitions is plotted and the result is deteriorated than (**a**).

Figure 20 shows the eye-diagram measurement results. Similar to the frequency domain measurement shown in Figure 19a, channels without and with TGV transitions are targeted. PRBS of $2^{8}-1$, with a rise-and-fall time of 30 ps, and an input data rate that corresponds to the (0,1) mode frequency (highlighted in Figure 19) is injected to port 1 and 3. In Figure 20a, a measured eye-diagram at port 2 is plotted. As a comparison, the measured eye-diagram at port 4 is plotted. At this data rate (corresponds to (0,1) PDN mode frequency), insertion loss of the channel increases due to the return current loading to the PDN. As a result, the eye-opening voltage is reduced, and the timing jitter increased. At the higher PDN resonance frequency, signal quality is deteriorated both in the frequency and the time domain. 

**Figure 21:**
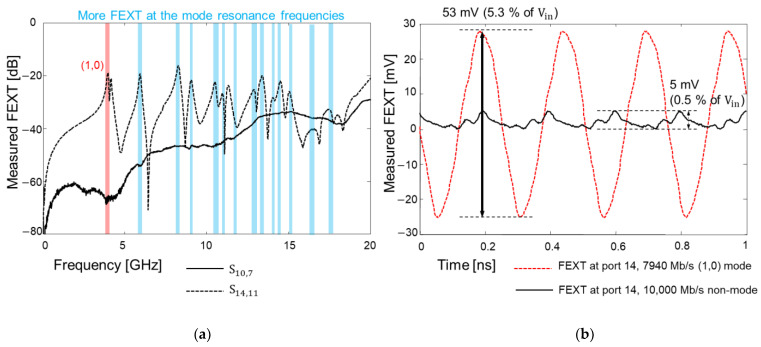
Measured far-end crosstalk (FEXT) comparison in the frequency domain (**a**) and in the time domain (**b**) are shown. When the signal TGV penetrates the PDN, it loads power/ground noise to the PDN and generates PDN induced FEXT.

The far-end cross-talk (FEXT) in the frequency domain and the time domain was analyzed by measuring the test vehicle shown in Figure 18b. Usually, FEXT impacts are minimized when the spacing between the adjacent channel is three times larger than the channel width [27]. The distance between coupled channels shown in Figure 18b is more than 30 times larger than the interposer channel width in M1 and M4 layer. Therefore, in this study, FEXT impacts should be both minimal in the frequency domain and the time domain. However, as can be seen from Figure 21a, the measured FEXT increased at certain frequencies when the interposer channel included via transition penetrating the PDN. In such a case, the signal TGV interacts with the PDN, and it is affected by the PDN mode resonances. Due to the high PDN impedance at the mode resonance frequencies, return current discontinuity occurs and the loaded return current propagates along the PDN. The loaded return current from the signal TGV serves as a power/ground noise wave in the PDN, propagates and couples to other channel structures. As a result, PDN-induced crosstalk occurs and becomes severe for the low-loss substrate. 

The time domain FEXT measurement results are plotted in Figure 21b. 7940 Mb/s clock signal which corresponds to the (1,0) PDN mode resonance frequency is injected to port 11 and the FEXT voltage is measured at port 14. The clock swings from 0 to 1 V with a 30 ps rise-and-fall time, and all ports are terminated with 50-ohm. In this case, 53 mV FEXT voltage is observed (5.3% of the input voltage). This value is 10 times larger than the case where the input, with a data rate that corresponds to 10,000 Mb/s (non-mode resonance frequency), clock signal is injected.

In this subsection, the impacts of the low loss substrate on the PDN impedance and return current loading are verified based on the measurements. Not only the activities of the circuit but also the signal via itself causes power/ground noise issues in the glass interposer PDN. In the following subsections, solutions are proposed and discussed to solve the power/ground noise issues in the glass interposer PDN.

### 4.3. Proposed Electromagnetic Bandgap Structures for a Broadband Noise Suppression

**Figure 22:**
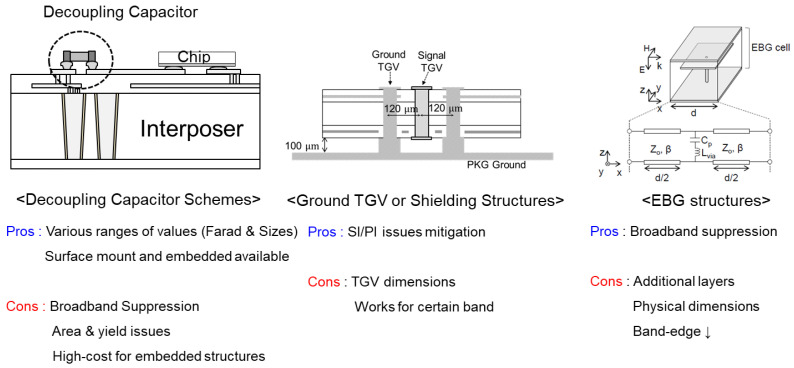
Pros and cons of each solution to solve power/ground noise issues are summarized.

There are various power/ground noise suppression methods in the field of packaging. Among the various solutions, decoupling capacitor schemes, electromagnetic bandgap (EBG) structures, and ground via array designs are widely used. As mentioned in earlier sections in this article, glass interposers are suitable for a broadband signaling. However, this means that the power/ground noise suppression must also be conducted in the same band. To achieve the broadband noise suppression using the decoupling capacitors, a large area in/on the interposer to assemble various decoupling capacitors with different values is required. The adoption of various decoupling capacitors will lead to yield issues due to the increased interposer area. An embedded package substrate (EPS) decoupling capacitor can be a solution, but it will cause a significant package/interposer cost increase [28]. Also, such solutions are still being developed for glass interposers, so it is currently immature. In Figure 22, the pros and cons of each solution are depicted. In this article, the EBG structures and TGV shields are focused on solving the power/ground noise issues in the glass interposer PDN. 

**Figure 23:**
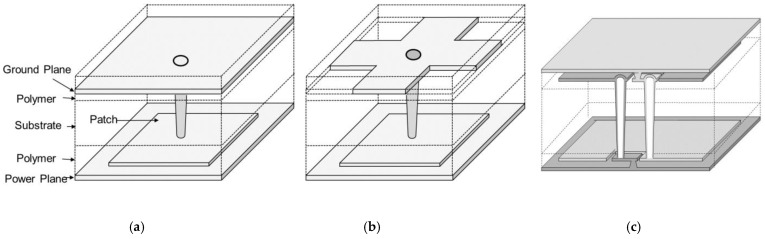
Designed EBG structures are depicted. Each structure is embedded in the glass interposer PDN, fabricated, and measured. (**a**) Mushroom type EBG which increases the capacitance of the PDN is shown. (**b**) Defected ground structure is added to the structure shown in Figure 23a. (**c**) Double-sided EBG structure which uses four layers is shown.

**Figure 24:**
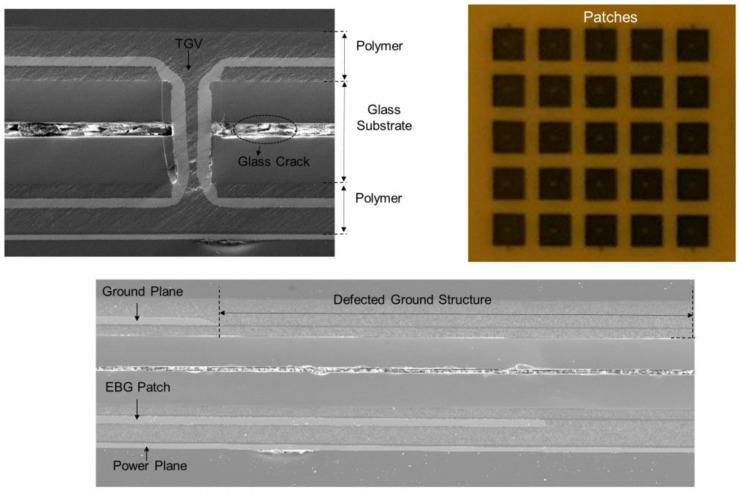
SEM and microscopic images of the fabricate structure is shown.

In Figure 23, the proposed and fabricated EBG structures, which can be embedded in the glass interposer PDN, are depicted. In Figure 23a, a mushroom type EBG structure is depicted. This structure is widely adopted in the packages and PCBs, but it was first adopted in previous work [29] at the interposer level. From this structure, structures are slightly modified to further broaden the noise suppression bandgap. In Figure 23b, defects are added in the ground plane. In Figure 23c, the double sided EBG structure is designed to provide more capacitance to the glass interposer PDN. To determine the suppression band, careful analysis must be conducted. The 3-D EM simulations predict the band accurately, but they require large computational resources and simulation times. In the previous work [13], an efficient method that predicts the suppression band based on a dispersion analysis in glass interposers is proposed and verified. In Figure 24, the SEM and microscopic images of the structure shown in Figure 23b are shown as representatives. These structures are fabricated and measured to verify the power/ground noise suppression characteristics.

**Figure 25:**
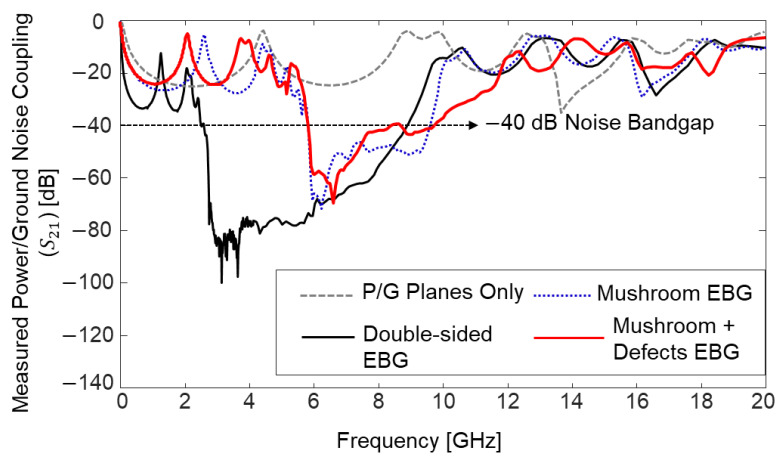
Measured power/ground noise couplings are compared.

**Figure 26:**
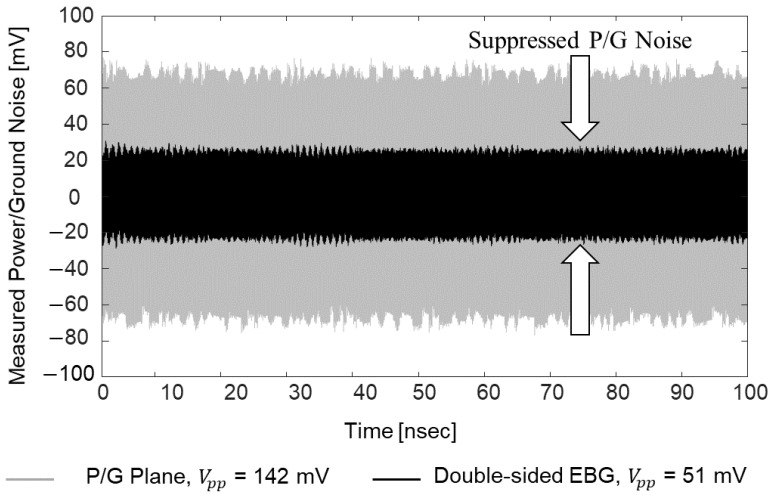
Power/ground noises are measured in the time domain for the verification.

Impacts of the proposed structures on power/ground noise suppression are verified in the frequency domain by comparing the power/ground noise coupling coefficient ($S_{21}$, but measurement ports are assigned within the same PDN). In Figure 25, measurement results are plotted and compared. By adopting the EBG structures, noise isolation/suppression (−40 dB) bandgaps are generated in the PDN. The effectiveness of the proposed structure is also validated in the time domain with measurements and simulations (EM + circuit). In Figure 26, measured power/ground noises in the PDN are compared. The PDN with power/ground planes only and the PDN with embedded double-sided EBG structure are compared. 

A pulse-pattern generator (PPG) (Anritsu MP-1763C) and a digital sampling oscilloscope (Tektronix TDS800B) were used to obtain the data. A 12 Gb/s clock signal (0 to 1 V, 30 ps rise-and-fall time, and all ports terminated with 50 ohm) was injected into the glass interposer PDN. The data rate of the noise induced to the PDN was in the noise suppression band of the proposed structure. It was approximately 15.5 mm from the noise source to the noise observation point which included the 5-unit structures shown in Figure 23c. Note that in Figure 23c, half of the unit structure is shown, whereas the structures shown in Figure 23a,b are a whole unit structure. Without the noise suppression structure, 142 mV peak-to-peak voltage ($V_{PP}$) is observed which is 14.2% of the input noise voltage. By adopting the proposed double-sided structure, the coupled noise is suppressed to 51 mV $V_{PP}$. The proposed structure suppressed and isolated power/ground noise in the PDN significantly.

**Figure 27:**
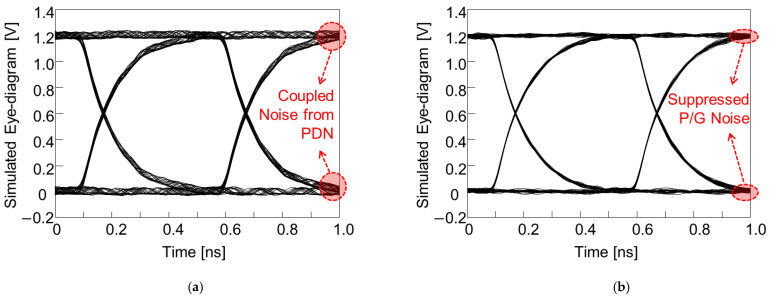
TGV channel escaping the PDN is simulated. (**a**) A case without the proposed EBG structure is plotted. (**b**) By adopting the proposed EBG structure, power/ground noise is suppressed.

In the 3-D EM simulator, the glass interposer PDN and TGV channel penetrating/escaping the PDN were designed and simulated. In this target study, the power/ground noise induced in the PDN propagates without attenuation due to low substrate loss, coupled with the TGV channel located far away from the noise source, and the SI of the TGV channel were degraded. The PRBS of 2^8^ − 1, 0 V to 1.2 V, with a rise-and-fall time of 30 ps, and data rate of 2 Gb/s was injected into the TGV channel affected by the power/ground noise. At the receiving side of the TGV channel where eye-diagrams are monitored, a capacitive termination (2 pF) was set. In Figure 27, eye-diagrams of the TGV channel without and with EBG structures in the PDN are compared. 

In this subsection, power/ground noise issues in the glass interposer PDN were measured and simulated. As a solution, EBG structures were designed, fabricated, and measured. Depending on the design, EBG structures are capable of achieving broadband noise suppression. Especially, the structure adding more capacitance to the PDN increased the noise suppression bandgap toward lower frequency. Adding some defected structures can expand the bandgap toward a higher frequency range. However, adding excessive amounts of defected structures can cause return current issues due to a reduced area in the ground plane. Lastly, using TGV with larger diameter or parallel TGVs in the unit structure can increase the bandgap toward high-frequency range as well. 

### 4.4. Effectiveness of the TGV Shields

**Figure 28:**
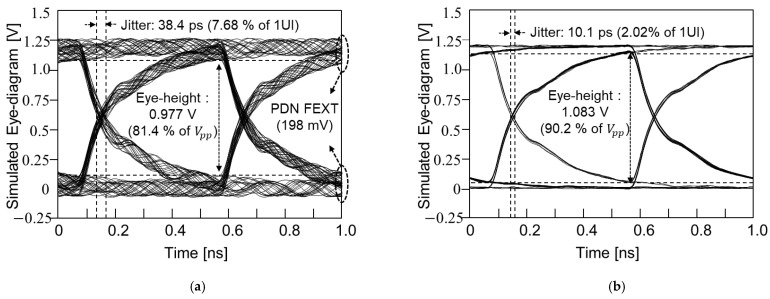
The impacts of ground TGV shields on power/ground noise suppression are shown. (**a**) Victim TGV channel without ground TGV shields is shown. (**b**) By adopting the ground TGV shield, impacts of power/ground noise is suppressed.

In this subsection, impacts of ground TGV design to shield the power/ground noise coupling are analyzed. The test vehicle shown in Figure 18b is simulated instead of measurement with ground TGV added adjacent to the signal TGV penetrating the PDN. A PRBS of 2^8^ − 1, 0 V to 1.2 V, with a rise-and-fall time of 30 ps, and data rate of 2 Gb/s was injected to the TGV channel (port 13). As an aggressor, an 8826 Mb/s clock signal (0 to 1 V, 30 s rise-and-fall time and all ports terminated with 50 ohm) which corresponds to the (1, 0)/(0, 1) mode resonance frequency was injected. Eye-diagrams were monitored at port 14 without and with the TGV shields. The received eye-diagram at port 14 had deteriorated severely due to the FEXT that propagated along the PDN. This result is shown in Figure 28a. By placing the ground TGV shields, FEXT induced from the PDN was suppressed and the result is shown in Figure 28b.

**Figure 29:**
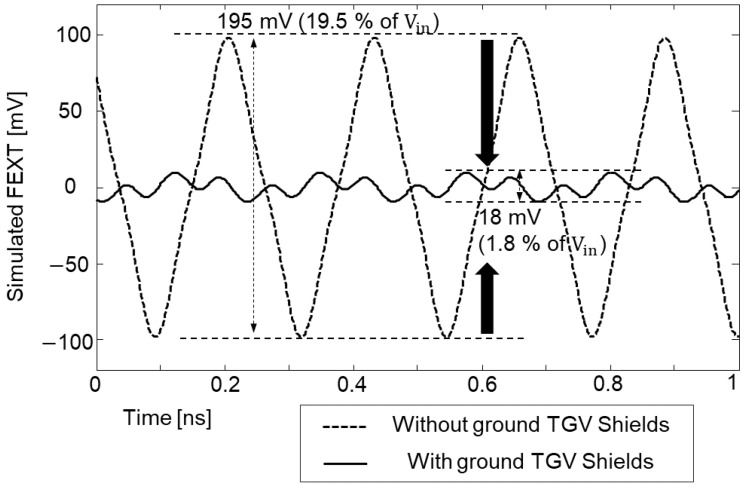
When the ground TGV is located near the aggressor, it also suppresses FEXT since it provides safer return current path for the aggressor, generating less power/ground noise loading.

It is also possible to suppress power/ground noise coupling in the glass interposer by shielding the noise source. Two ground TGVs near the aggressor TGV channels are located and simulated. The ground shield TGV also provided a return current path less affected by the PDN resonance for the aggressor which generated less power/ground noise loading from the signal TGV to the glass interposer PDN. Figure 29 shows the effectiveness of the ground TGV shield on the FEXT suppression when it is placed adjacent to the aggressor. The ground shield TGV is not only effective for shielding the noise coupling, but also effective for suppressing the noise near the source.

When designing the high-speed interconnection, including the TGV that penetrates the PDN, adopting the ground TGV to provide better return current path and to shield the power/ground noise is important. However, it is difficult to assign 1:1 or multiple ground TGVs to each signal TGV due to the limited area of the interposer. This is the same for designing packages, PCBs, or other interposers. Therefore, various simulations and analyses must be conducted before fabrication. 

## 5. Conclusions

In this article, an in-depth electrical performance analysis was provided based on various test vehicle measurements and simulations to fully benefit the advantages of the low-loss glass substrate. Various results from previous works are also reviewed to emphasize the advantages. Also, issues that must be solved were delivered and verified. 

First, the fabrication process and test vehicles for the analysis are explained. In the following section, using the test vehicles, the electrical performance of the glass interposer’s high-speed interconnection was compared with those of silicon and organic interposers. The insertion loss, eye-diagram, and signal bandwidth of three interposer channels were compared and analyzed to validate the advantages of the glass interposer. Frequency domain simulation results, eye-diagram simulations, and analysis in the time domain were conducted. The high-speed interconnection in the glass interposer showed a larger eye-opening voltage and smaller timing jitter than other interposers. Capacitance and conductance of the glass and silicon channels to determine factors affecting the loss profile were thoroughly analyzed. The conductivity of the silicon substrate contributed to the high insertion loss of the high-speed interconnection in the silicon interposer. This high conductivity also affected the via-to-via noise coupling. The via-to-via noise coupling between TSV and through glass via (TGV) was compared and measured using the fabricated test vehicles. Overall, the high-speed interconnection in the glass interposer showed superior electrical performance than silicon and organic interposers.

In spite of the channel properties associated with the low substrate loss, this low loss causes problems when the noise is generated in the glass interposer PDN. To define and prove the power/ground noise issues, various cases inducing the power/ground noise in the PDN were considered, simulated, and measured. Impedance profiles of the interposer PDNs are compared for three substrates. The noise propagation and coupling issues were also compared and analyzed. The glass interposer PDN is severely deteriorated by the noise in the PDN. To solve the issues, ground TGV design and electromagnetic bandgap (EBG) design are proposed for an efficient broadband suppression of the noise generated in the glass interposer PDN. 

The glass interposer has many advantages in terms of cost reduction and superior channel characteristics. However, in order to fully take advantage of the glass interposer, power/ground noise suppression must be conducted. In this article, EBG structures and ground TGV shields are proposed. The effectiveness of the proposed structures is verified by measurements and simulations. Since the fabrication process keeps improving, in the near future, the glass interposer can be a superior alternative to the current silicon interposer for 2.5-D system integration and scaling. 

## Figures and Tables

**Table 1:** Occurrence probability equation summary when DBI coding is adopted.

	Symbol	Value
Physical Dimensions	*h_glass_*	100 μm
	*t_pol1_*	35 μm
	*t_pol2_*	17.5 μm
	*t_m_*	10 μm (7 for μm M4)
	*t_TGV_*	Apr. 12~15 μm
	*d_TGVT_*	100 μm
	*d_TGVB_*	60 μm
	*d_padTGV_*	120 μm
	$d_{\mu via}$	45 μm
	$d_{pad_{\mu via}}$	75 μm
Material Properties	ε *_glass_*	5.3 at 2.4 GHz
	ε *_pol_*	3 at 10 GHz
	*tan* δ *_glass_*	0.004 at 2.4 GHz
	*tan* δ *_pol_*	0.005 at 10 GHz
	σ *_m_*	5.8 $\times \;10^{7}$σ/m

**Table 2:** Physical dimensions and material properties of the silicon and organic interposers.

	Symbol	Value	
		Silicon	Organic
Physical Dimensions	*W*	10 μm	10 μm
	*S*	10 μm	10 μm
	*Substrate, build-up layer, metal heights are identical to the values shown in* *Table 1*		
Material Properties	ε _rsub_	11.2	4.4
	ε _r1_	4.0	4.4
	ε _r2_	4.0	4.4
	$tan\;\delta_{sub}$	-	0.02
	$tan\;\delta_{1}$	0.012	0.02
	$tan\;\delta_{2}$	0.012	0.02
	$\sigma_{sub}$	10	-
	$\sigma_{metal}$	$5.8\times 10^{7}$	$5.8\times 10^{7}$

## Appendix A. 3-D Electromagnetic (EM) Simulation Conditions

In this appendix, 3-D EM simulation conditions are summarized for readers. When assigning the radiation boundary, an air box is generated with 50% larger in ±x and ±y dimensions of the DUT and 300% larger in ±z dimension of the DUT. Lumped ports are assigned for all simulations. For signal ports, rectangles with the same width as the target signal channel are assigned in between signal and ground interconnects. For PDN ports, rectangles with 50 μm in width are assigned in between power and ground interconnects. For the channel bandwidth analysis, simulations are conducted up to 100 GHz. Other simulations are conducted up to 20 GHz. If simulated under the linear frequency-step condition, it is difficult to obtain an accurate result under 1 GHz. Therefore, a logarithmic step condition is applied for all analysis.
